# The urokinase receptor/uPAR is a major effector of p53 gain-of-function mutations in gemcitabine-treated pancreatic ductal adenocarcinoma

**DOI:** 10.1016/j.jbc.2025.110561

**Published:** 2025-08-07

**Authors:** Carlotta Zampieri, Kourosh Kouhmareh, Parnian Sartipdolagh, Pardis Azmoon, Richard L. Klemke, Steven L. Gonias

**Affiliations:** Department of Pathology, University of California San Diego School of Medicine, La Jolla, California, USA

**Keywords:** urokinase receptor, p53, plasminogen activator, cancer therapy, chemoresistance

## Abstract

In pancreatic ductal adenocarcinoma (PDAC), chemotherapy may select for cancer cells with increased capacity for invasion and metastasis. The responsible mechanisms remain incompletely elucidated; however, *TP53*, which carries gain-of-function (GoF) mutations, has been implicated. *PLAUR* encodes a cellular receptor, uPAR, which promotes cancer invasion, metastasis, epithelial–mesenchymal transition (EMT), and resistance to chemotherapy. Mining TCGA showed that in PDAC, *PLAUR* expression is substantially increased and the extent of increase correlates with cancer stage. High *PLAUR* expression is associated with decreased overall, disease-free, and progression-free survival. *PLAUR* expression is increased in PDACs with *TP53* GoF mutations. In PANC1 and MIA PaCa-2 PDAC cells, which express *TP53* GoF mutations, gemcitabine treatment increased *PLAUR* expression, ERK1/2 activation, and ribosomal S6 kinase phosphorylation in surviving cells. The surviving cells also demonstrated increased migration and invasion. The increase in uPAR abundance was sustained and continued to increase after gemcitabine was withdrawn. Silencing *TP53* blocked the gemcitabine-induced increase in uPAR abundance and the associated increases in ERK1/2 activation, cell migration, and invasion. Silencing *EGFR* did not affect uPAR-promoted ERK1/2 activation, despite the known ability of the EGF Receptor to collaborate with uPAR in activating cell signaling under select circumstances. scRNA-Seq of human PDACs showed that *PLAUR* expression is increased in specific clusters of malignant epithelial cells post-treatment. High *PLAUR* expression is associated with increased basal cell-like gene expression signatures, copy number instability, and EMT signatures. Mutated *TP53*-induced *PLAUR* expression represents a major pathway by which PDAC treatment may lead to chemotherapy resistance and increased cancer cell aggressiveness.

Pancreatic ductal adenocarcinoma (PDAC) is an aggressive cancer, with an estimated incidence of 13/100,000 people and a 5-year survival rate below 10% (1). Rapid progression and lack of early detection contribute to the negative prognosis. The ability of PDAC cells to acquire resistance to anticancer drugs is another major challenge (2). Understanding the mechanisms by which PDAC cells acquire resistance to chemotherapy is an essential goal.

PDAC development is facilitated by mutations in *TP53* (3). The protein product of wild-type *TP53*, p53, has been referred to as the “guardian of the genome” because of its ability to function as a tumor suppressor, regulate DNA repair, and promote cell cycle arrest in response to genetic damage (4, 5). However, in cancers, missense mutations in *TP53* profoundly alter its function, not only eliminating anticancer functions but also conferring oncogenic properties and promoting progression of established cancers. The cancer-promoting properties of mutated *TP53* are referred to as “gain of function” (GoF) activities (6). Molecular mechanisms that underlie p53 GoF activities remain incompletely understood.

*TP53* GoF mutations are linked to cancer chemotherapy resistance in PDAC (7, 8, 9). Silencing expression of *TP53* in PDAC cells attenuates resistance to gemcitabine, a commonly used PDAC chemotherapy drug. Transcriptome profiling of PDAC cells, before and after silencing of *TP53*, suggests that *TP53* GoF mutations drive extensive alterations in diverse processes, including chromatin remodeling and cellular metabolism (8, 10, 11, 12). Mutated p53 facilitates cancer cell survival, proliferation, and resistance to stress (13, 14). Mutated p53 also promotes NF-κB activation to upregulate expression of pro-inflammatory cytokines and chemokines (15). This interaction may foster the establishment of a tumor-promoting pro-inflammatory environment.

In this study, we uncovered a novel relationship between mutated *TP53* and uPAR, the receptor for urokinase-type plasminogen activator (uPA). Binding of uPA to uPAR promotes plasminogen activation and remodeling of the extracellular matrix (16, 17). The same interaction also activates cell-signaling pathways, reflecting the activity of a multiprotein receptor complex that includes, in addition to uPAR, FPR1, receptor tyrosine kinases, and integrins (18, 19, 20). In normal tissues, uPAR expression is limited (21); however, in cancers, the activities of uPAR are exploited to promote invasion and metastasis, survival under stress, epithelial–mesenchymal transition (EMT), and expression of stem cell-like properties (22, 23, 24, 25, 26, 27, 28). uPAR has been shown to be responsible for acquired resistance to specific anticancer agents (29, 30, 31).

The results presented herein show that in PDAC cells with *TP53* GoF mutations, expression of the gene encoding uPAR (*PLAUR*) is substantially increased by treatment with gemcitabine. uPAR activates ERK1/2, a known cell survival event (22, 32, 33), and promotes migration and invasion of PDAC cells. Silencing *TP53* blocks uPAR expression in response to gemcitabine. The ability of uPAR to function as a major effector of mutated *TP53*, in treated human PDACs, was confirmed by analysis of TCGA and scRNA-Seq data from human PDACs. Targeting the p53-uPAR axis may be an effective strategy to prevent chemotherapy resistance and increased PDAC cell aggressiveness in response to chemotherapy.

## Results

### PLAUR expression is increased when TP53 is mutated in PDAC

**Figure 1:**
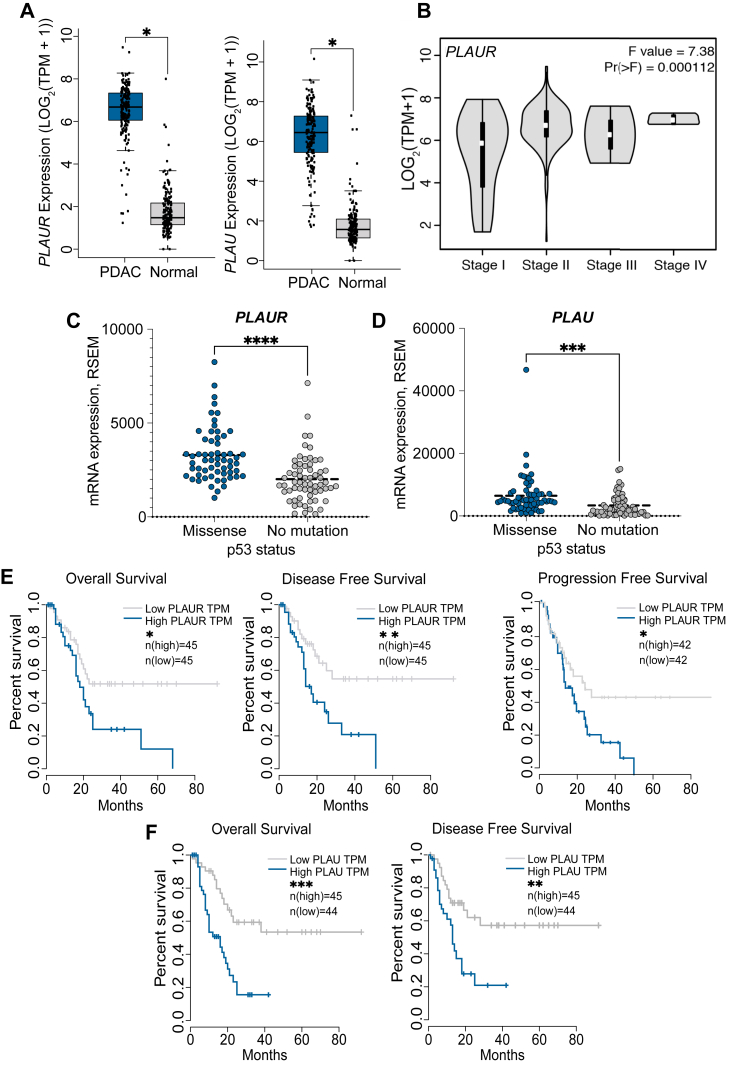
***PLAUR* and *PLAU* are highly expressed in PDAC, correlating with *TP53* mutation and poor prognosis**. *A*, *PLAUR* and *PLAU* mRNA expression (Transcripts per million, TPM) in PDAC tissues (n = 179) compared with adjacent normal tissues (n = 171). *p*-values were calculated using a one-way ANOVA (∗*p* < 0.05; data source: GEPIA). *B*, Violin plots showing *PLAUR* mRNA expression (TPM) across different clinical stages of PDAC (Stages I–IV; one-way ANOVA; data source: GEPIA). *C and D*, Scatter dot plots illustrating *PLAUR* (*C*) and *PLAU* (*D*) mRNA expression (RSEM, batch normalized from Illumina HiSeq_RNASeqV2) in human PDACs stratified by *TP53* mutation status. Patients harboring *TP53* missense mutations (n = 60 for *PLAUR*; n = 62 for *PLAU*) were compared with patients lacking *TP53* mutations (n = 63 for *PLAUR*; n = 66 for *PLAU*). Statistical analysis was performed using unpaired two-tailed t-tests (∗∗∗*p* < 0.001; ∗∗∗∗*p* < 0.0001; data source: TCGA PanCancer Atlas *via* cBioPortal). *E and F*, Kaplan–Meier survival curves showing OS, DFS, and PFS in PDAC patients from the TCGA PanCancer Atlas dataset (cBioPortal). Patients were stratified into quartiles based on *PLAUR* (*E*) or *PLAU* (*F*) mRNA expression. Statistical significance was determined by log-rank Mantel-Cox tests (∗*p* < 0.05; ∗∗*p* < 0.01; ∗∗∗*p* < 0.001).

We mined TCGA using the GEPIA platform (Gene Expression Profiling Interactive Analysis RRID:SCR_018294) to compare the expression of *PLAUR* mRNA in PDAC tumor tissue *versus* adjacent normal tissue. Figure 1*A* shows that *PLAUR* expression was significantly higher in tumor tissue. Expression of the gene encoding uPA (*PLAU*) was also increased in PDAC tumor tissue compared with adjacent normal tissue. *PLAUR* gene expression increased progressively in primary tumors as the stage of PDAC increased (Fig. 1*B*).

Mutations in *TP53* are observed in PDAC and high-grade pancreatic intraepithelial neoplasia (3). *TP53* missense mutations convey GoF activities, which promote cancer progression (34). Analysis of a cohort of 184 PDAC patients in TCGA demonstrated that patients with *TP53* GoF mutations exhibited significantly higher *PLAUR* expression compared with those with wild-type *TP53* (Fig. 1*C*). *PLAU* expression was also higher in PDAC samples with *TP53* missense mutations (Fig. 1*D*). These results are important because increased *PLAUR* expression in PDAC is associated with poorer overall survival (OS), disease-free survival (DFS), and progression-free survival (PFS) (Fig. 1*E*). Similarly, increased *PLAU* expression is associated with decreased OS and DFS (Fig. 1*F*). Previous analyses of distinct tissue specimens have also demonstrated an inverse relationship between *PLAUR* expression and patient survival (35, 36).

### Gemcitabine treatment of PDAC cells increases uPAR protein levels

**Figure 2:**
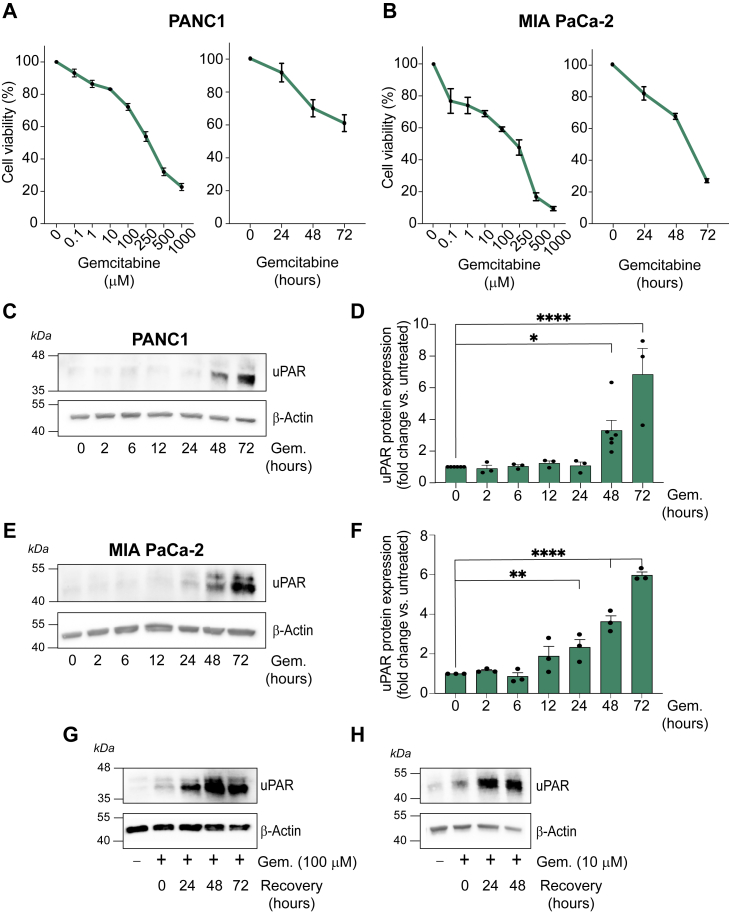
**uPAR protein abundance is increased in PDAC cell lines following gemcitabine treatment.***A and B*, MTT analysis of cell viability in response to gemcitabine treatment in PANC1 and MIA PaCa-2 cells. *A*, PANC1 cells were treated with increasing concentrations of gemcitabine (0.1–1000 μM) for 48 h (*left* panel; n = 3; mean ± SEM) or with 100 μM gemcitabine for 24, 48 and 72 h (*right* panel; n = 6; mean ± SEM). *B*, MIA PaCa-2 cells were treated with increasing concentrations of gemcitabine (0.1–1000 μM) for 48 h (*left* panel; n = 4–6; mean ± SEM) or with 10 μM gemcitabine for 24, 48 and 72 h (*right* panel; n = 6; mean ± SEM). *C–F*, time-course analysis of uPAR protein levels in response to gemcitabine. Immunoblot analysis of uPAR protein in PANC1 (*C* and *D*) and MIA PaCa-2 cells (*E* and *F*) treated with gemcitabine (100 μM for PANC1; 10 μM for MIA PaCa-2). Cells were harvested at indicated time points (0, 2, 6, 12, 24, 48, and 72 h). Blots were probed for uPAR and β-actin as a loading control. Densitometric quantification (*D* and *F*) was performed with ImageJ. uPAR protein abundance was normalized to β-actin and expressed as the fold change relative to the 0-h baseline. Data represent the mean ± SEM of 3 to 4 independent experiments; statistical significance was determined by one-way ANOVA analysis with Dunnett's multiple comparisons test (∗*p* < 0.05; ∗∗*p* < 0.01; ∗∗∗*p* < 0.001; ∗∗∗∗*p* < 0.0001). *G and H*, Changes in uPAR protein abundance following gemcitabine removal. Immunoblot analysis of uPAR protein abundance in PANC1 (*G*) and MIA PaCa-2 (*H*) cells that were treated with gemcitabine (100 μM and 10 μM, respectively) for 48 h and then allowed to recover in the absence of gemcitabine for the indicated time periods (24, 48, and 72 h for PANC1 cells; 24 and 48 h for MIA PaCa-2 cells). Immunoblots were re-probed for β-actin as a loading control. Each experiment was repeated three times.

PANC1 cells harbor the *TP53* gain-of-function mutation, R273H. p53 R273H is a common missense mutation in PDAC, associated with poor outcomes (8, 12, 37, 38). To test the effects of gemcitabine on PANC1 cell survival, we treated PANC1 cells with gemcitabine (0.1–1000 μM) for 48 h. These cells were only modestly responsive to the antitumor drug (Fig. 2*A*); 100 μM gemcitabine decreased the number of viable cells by 28 ± 2% (n = 3). Next, PANC1 cells were treated with 100 μM gemcitabine for different times, up to 72 h. Although gemcitabine induced time-dependent cell death, at 72 h, 61 ± 2% (n = 6) of the cells remained. The relative chemoresistance of PANC1 cells has been previously reported (8).

The response of MIA PaCa-2 cells to gemcitabine is shown in Figure 2*B*. MIA PaCa-2 cells harbor the *TP53* R248W mutation, which has been shown to exert GoF activities (37, 38). In dose–response studies, treatment of MIA Paca-2 cells with 10 μM gemcitabine for 48 h was sufficient to decrease the number of viable cells by 31 ± 2% (n = 3). 73 ± 1% (n = 6) of the viable cells were eliminated by 10 μM gemcitabine at 72 h. Thus, although MIA PaCa-2 cells were more susceptible to gemcitabine, viable populations remained after gemcitabine treatment.

Next, we examined uPAR protein levels in gemcitabine-treated and control PANC1 cells by immunoblot analysis. In the absence of gemcitabine, uPAR protein expression was modest and required extended immunoblot exposure times to be detected. However, when treated with 100 μM gemcitabine for 48 to 72 h, uPAR protein abundance increased (Fig. 2*C*). The results of three independent experiments are summarized in Figure 2*D*.

MIA PaCa-2 cells also expressed limited amounts of uPAR protein in the absence of gemcitabine, but within 24 h of treatment with 10 μM gemcitabine, the abundance of uPAR increased (Fig. 2*E*). Densitometry results from three separate experiments showed that the increase in uPAR abundance was significant at 24 to 72 h (Fig. 2*F*).

To determine whether the increase in uPAR protein abundance induced by gemcitabine is stable when the chemotherapy agent is removed, we cultured PANC1 cells in the presence of 100 μM gemcitabine for 48 h and then transferred the cells to fresh medium without gemcitabine. Figure 2*G* shows that uPAR was not only stable but continued to increase over the next 24 to 72 h. Similar results were obtained with MIA PaCa-2 cells (Fig. 2*H*). These results show that increased uPAR expression is maintained by PDAC cells after chemotherapy is terminated.

### TP53 GoF mutations drive gemcitabine-induced uPAR upregulation in PDAC cells

**Figure 3:**
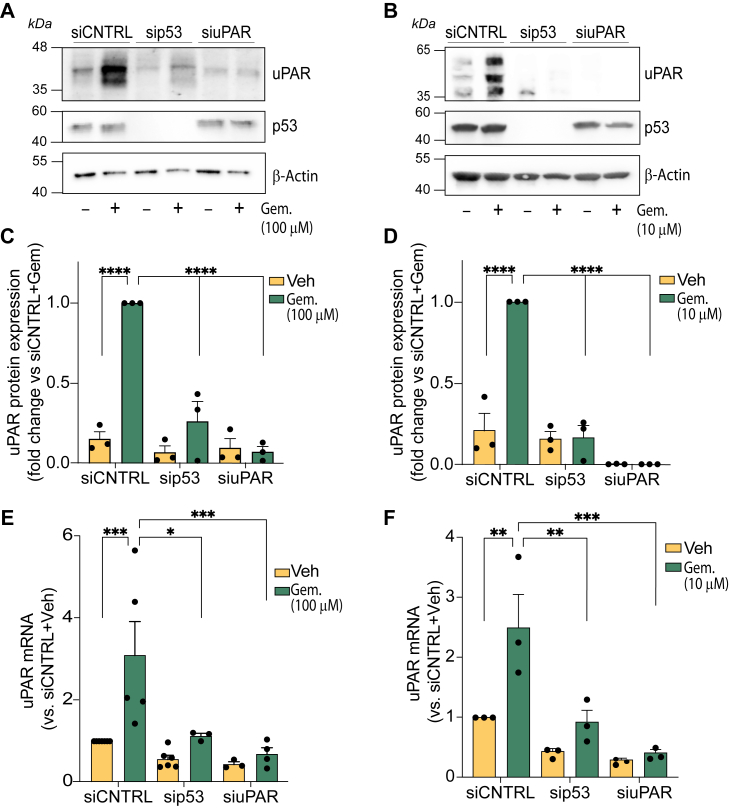
**p53 controls expression of uPAR protein and mRNA**. The relationship between p53 and uPAR was evaluated in PANC1 and MIA PaCa-2 cells. Cells were transfected with siRNA targeting *TP53*, *PLAUR*, or with control siRNA (siCNTRL) for 24 h and then treated with gemcitabine (100 μM for PANC1 cells and 10 μM for MIA PaCa-2 cells) for 72 h. *A–D*, immunoblot analysis showing uPAR protein expression in PANC1 (*A* and *C*) and Mia PaCa-2 (*B* and *D*) cells; blots were re-probed for β-actin as a control for load. Densitometric analysis (*C* and *D*) was performed using ImageJ. uPAR abundance was normalized using β-actin and expressed as the fold change relative to the level observed in siCNTRL-transfected cells treated with gemcitabine. The results represent three independent experiments (mean ± SEM; one-way ANOVA and Dunnett's multiple comparisons test; ∗∗∗∗*p* < 0.0001). *E and F*, uPAR mRNA levels in PANC1 (*E*) and MIA PaCa-2 (*F*) cells were measured by RT-qPCR. Bars represent the mean ± SEM from three independent experiments. Statistical significance was determined by one-way ANOVA with Dunnett's test (statistical comparison *versus* siCNTRL + Gem; ∗∗*p* < 0.01; ∗∗∗*p* < 0.001; ∗∗∗∗*p* < 0.0001).

siRNAs were used to silence the expression of *TP53* or *PLAUR* in PANC1 cells. Figure 3*A* shows that 72 h after transfection, *TP53* silencing was nearly 100% effective. Cells that were transfected with Silencer Select Negative Control No. 1 siRNA (siCNTRL) demonstrated the anticipated increase in uPAR protein abundance after treatment with 100 μM gemcitabine for 72 h. The effects of gemcitabine on uPAR protein abundance were blocked by *PLAUR*-specific siRNA, as anticipated. Silencing *TP53* also blocked the gemcitabine-induced increase in uPAR protein abundance.

Equivalent experiments were performed with MIA PaCa-2 cells (Fig. 3*B*). Gemcitabine treatment for 72 h increased the abundance of uPAR protein in cells treated with siCNTRL. This response was blocked in cells transfected with *PLAUR*-specific siRNA or *TP53*-specific siRNA. Densitometry analysis of three independent experiments showed that the effects of *PLAUR*-specific and *TP53*-specific siRNA on uPAR abundance following gemcitabine treatment were statistically significant in both PANC1 (Fig. 3*C*) and MIA PaCa-2 cells (Fig. 3*D*).

To confirm the role of p53 in mediating the increase in *PLAUR* expression in gemcitabine-treated PDAC cells, we measured *PLAUR* mRNA by RT-qPCR. In PANC1 cells (Fig. 3*E*) and MIA PaCa-2 cells (Fig. 3*F*), *PLAUR*-specific siRNA almost completely blocked the gemcitabine-induced increase in *PLAUR* mRNA, as anticipated. *TP53*-specific siRNA also significantly decreased gemcitabine-induced *PLAUR* mRNA expression.

### uPAR promotes ERK1/2 activation in PDAC cells in response gemcitabine

The MAPK/ERK1/2 signaling pathway regulates key cancer-related processes, including survival, proliferation, metastasis, and chemoresistance (32, 39). Aberrant ERK1/2 activation has been implicated in tumor progression (40). uPAR functions as an activator of ERK1/2 in multiple malignancies, including breast cancer and glioblastoma (22, 26, 29). We tested whether gemcitabine-induced uPAR expression in PDAC cells results in activation of ERK1/2.

**Figure 4:**
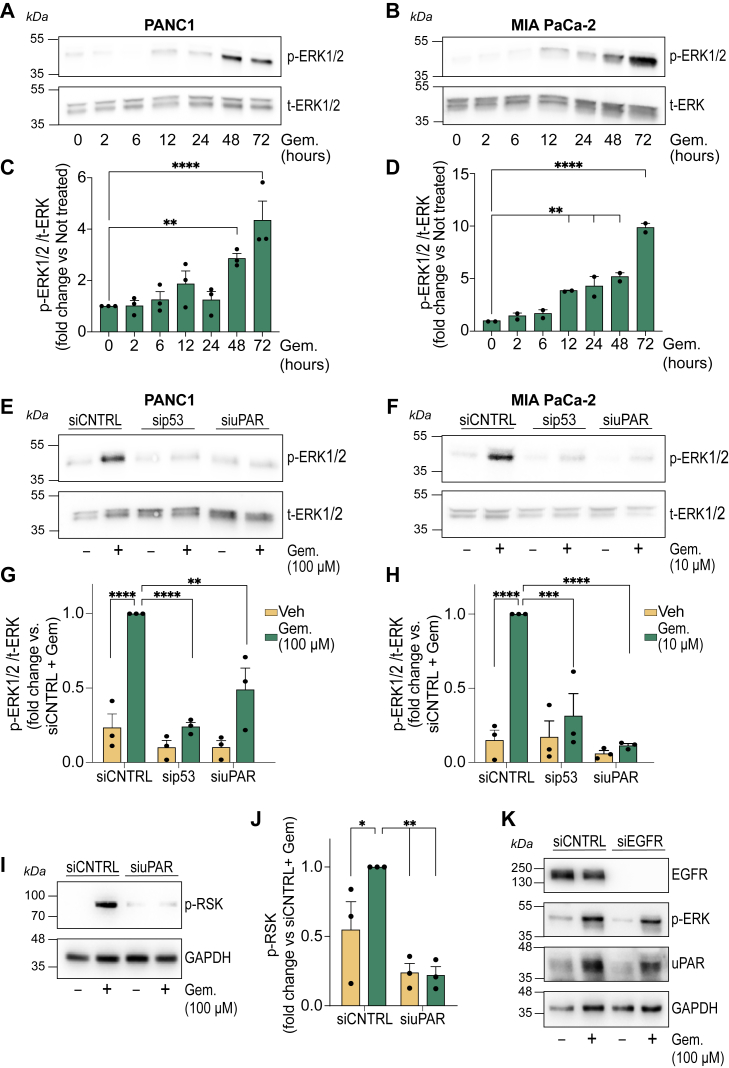
**uPAR promotes ERK1/2 and RSK activation in PDAC cells treated with gemcitabine**. *A–D*, PANC1 (*A* and *C*) and MIA PaCa-2 (*B* and *D*) cells were exposed to gemcitabine (100 μM for PANC1 cells; 10 μM for MIA PaCa-2 cells). Cell extracts were collected at 0, 2, 6, 12, 24, 48, and 72 h. p-ERK1/2 and t-ERK1/2 levels were assessed by immunoblotting (*A* and *B*). Quantification of p-ERK1/2, normalized to t-ERK1/2, was performed and expressed as the fold change relative to the 0-h baseline (*C* and *D*). Results are presented as the mean ± SEM of three independent experiments (one-way ANOVA and Dunnett's test; ∗∗*p* < 0.01; ∗∗∗∗*p* < 0.0001). *E–H*, ERK1/2 activation following *TP53* or *PLAUR* silencing. PANC1 (*E* and *G*) and MIA PaCa-2 (*F* and *H*) cells were transfected with siRNAs targeting *TP53* or *PLAUR* or with control siRNA, and 24 h later, treated with gemcitabine (100 μM for PANC1 cells; 10 μM for MIA PaCa-2 cells) for 72 h. Immunoblot analysis was performed to compare p-ERK1/2 and t-ERK1/2 levels (*E* and *F*). Three independent studies were performed and subjected to densitometry (*G* and *H*). Results are expressed as the mean ± SEM (standardized to samples transfected with siCNTR and treated with gemcitabine; one-way ANOVA and Dunnett's test; ∗∗*p* < 0.01; ∗∗∗*p* < 0.001; ∗∗∗∗*p* < 0.0001). *I*, immunoblot analysis of p-RSK in PANC1 cells treated with 100 μM gemcitabine for 72 h, following transfection with siCNTRL or siRNA targeting *PLAUR*. GAPDH was examined as a loading control. *J*, Densitometric quantification of p-RSK levels normalized to GAPDH in three independent experiments. Data represent the mean ± SEM (∗∗*p* < 0.01, ∗*p* < 0.05; compared to siCNTRL + Gemcitabine, one-way ANOVA and Dunnett's test). *K*, immunoblot analysis of EGF-R, uPAR, and p-ERK1/2 in PANC1 cells transfected with siRNA targeting *EGFR* or with siCNTRL and subsequently treated with 100 μM gemcitabine for 72 h. The results shown are representative of three independent experiments.

PANC1 cells were treated with 100 μM gemcitabine for up to 72 h. ERK1/2 phosphorylation was increased by 48 h (Fig. 4*A*). Similar results were observed with MIA PaCa-2 cells treated with 10 μM gemcitabine (Fig. 4*B*). Densitometry analysis showed that the observed increases in phospho-ERK1/2 were statistically significant in PANC1 cells (Fig. 4*C*) and MIA PaCa-2 cells (Fig. 4*D*).

The gemcitabine-induced increase in phospho-ERK1/2 was replicated in PANC1 cells and MIA PaCa-2 cells transfected with siCNTRL (Fig. 4, *E* and *F*). By contrast, *TP53*-targeting siRNA and *PLAUR*-targeting siRNA blocked the increase in ERK1/2 phosphorylation caused by gemcitabine. Densitometry analysis showed that the results were statistically significant (Fig. 4, *G* and *H*). The effects of *PLAUR* silencing on ERK1/2 phosphorylation in gemcitabine-treated cells were confirmed using a *PLAUR*-targeting pooled siRNA product (Fig. S1, *A* and *B*). These findings support the hypothesis that uPAR drives the increase in phospho-ERK1/2, observed in gemcitabine-treated PDAC cells with *TP53* mutations.

Ribosomal S6 kinases (RSK1/2/3) are downstream targets of activated ERK1/2, which, when activated, promote cell survival (41, 42). Figure 4*I* shows that gemcitabine treatment increased phospho-RSK (p-RSK) in PANC1 cells. Analysis of three separate experiments showed that *PLAUR* silencing significantly inhibited the gemcitabine-induced increase in p-RSK (Fig. 4*J*).

The EGF Receptor (EGF-R) has been implicated in uPAR-initiated cell-signaling although uPAR-signaling may occur independently of the EGF-R as well (43, 44, 45). We tested whether the EGF-R is required for uPAR-facilitated ERK1/2 activation in PANC1 cells by silencing *EGFR*. Figure 4*K* shows that *EGFR* silencing was highly effective in decreasing the abundance of EGF-R protein; however, in three separate experiments, *EGFR* silencing did not affect the gemcitabine-induced increase in uPAR protein expression or the resulting increase in ERK1/2 phosphorylation. These results suggest that in gemcitabine-treated PDAC cells, uPAR-mediated ERK1/2 activation may occur independently of the EGF-R.

### uPAR promotes cell migration and invasion in gemcitabine-treated PDAC cells

**Figure 5:**
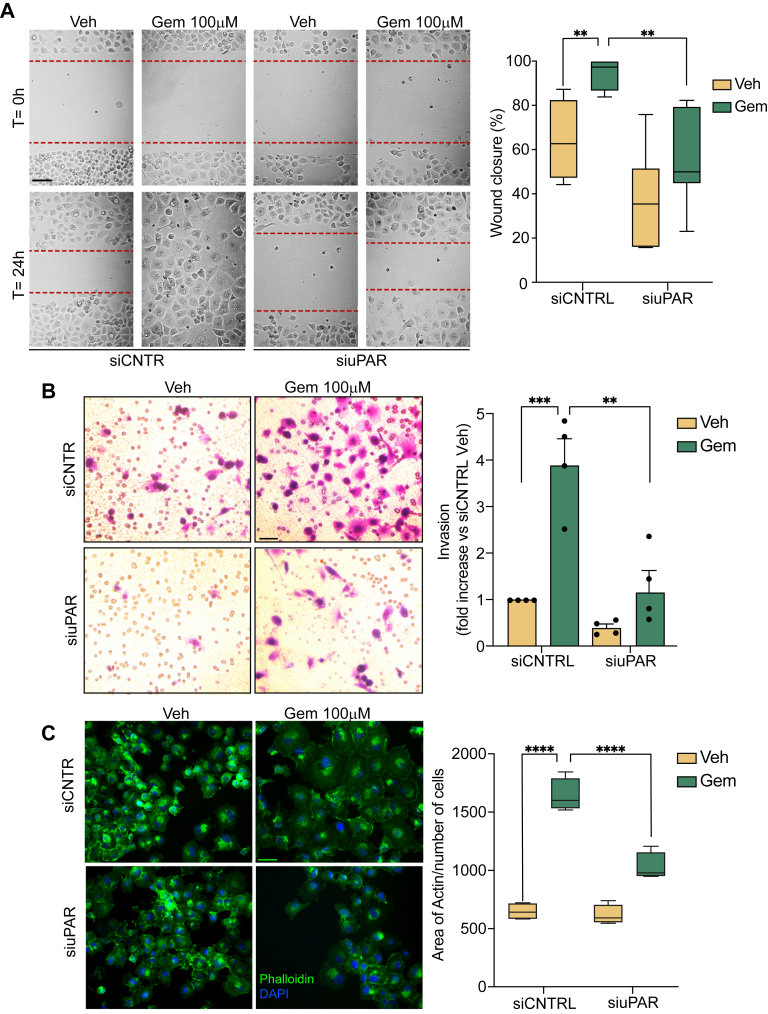
***PLAUR* silencing inhibits gemcitabine-induced cell migration, invasion, and enlargement of adherent PANC1 cells**. PANC1 cells were transfected with siRNA targeting *PLAUR* or with siCNTRL for 24 h. *A*, cells were seeded into cell culture wells with silicone culture inserts to create a defined gap for migration assays and treated with 100 μM of gemcitabine for 48 h. The inserts were removed and the cells allowed to migrate into the gap for 24 h. Representative images were captured at 0 and 24 h and the percentage of wound closure was quantified. Results represent the mean ± SEM from seven independent experiments (∗∗*p* < 0.001; one-way ANOVA and Dunnett's test; scale bar = 100 μm). *B*, cell invasion was assessed. After transfection with siRNA, PANC1 cells were treated with 100 μM gemcitabine for 48 h. Cells then were seeded into Geltrex Matrix-coated Transwell inserts and allowed to invade for 36 h. Invaded cells were fixed, stained, and counted. Results are normalized to those obtained with untreated cells transfected with siCNTRL (mean ± SEM of four independent experiments; ∗∗*p* < 0.01; ∗∗∗*p* < 0.001; two-way ANOVA and Tukey test; scale bar = 100 μm). *C*, to assess cell size, PANC1 cells were transfected with siRNA and treated with gemcitabine (100 μM) or vehicle for 48 h. Cells were then fixed and stained with Phalloidin, followed by imaging on a Leica DMi8 microscope. The area of phalloidin-stained actin in each cell was quantified using ImageJ. Mean cell size was calculated by dividing total actin area by cell number (scale bar = 50 μm; the results shown are the mean ± SEM from four independent experiments; one-way ANOVA and Dunnett's test; ∗∗∗∗*p* < 0.0001).

We examined migration of PANC1 cells (46) into cell-free areas of culture wells formed using plastic inserts (Fig 5*A*). Gemcitabine significantly increased PANC-1 cell migration, as determined by the more rapid closure of gaps. When *PLAUR* expression was silenced, gemcitabine-induced cell migration was significantly inhibited. In PANC1 cells that were not treated with gemcitabine, *PLAUR* silencing showed a trend toward inhibiting cell migration; however, the results did not achieve statistical significance (*p* = 0.07).

Next, we examined PDAC cell invasion, using Geltrex matrix to model basement membrane barriers. Figure 5*B* shows that gemcitabine treatment significantly increased PANC1 cell invasion. The increase in cell invasion induced by gemcitabine was substantially inhibited by *PLAUR* gene silencing. The effects of gemcitabine on cancer cell migration and invasion have been demonstrated previously (47, 48). The role of uPAR as a mediator of this response is not previously reported.

uPAR regulates Rho family GTPases, which control cell adhesion, spreading, and migration (49, 50). To test whether gemcitabine affects adhesion and spreading of PANC1 cells, adherent cells were treated with 100 μM gemcitabine and then stained with phalloidin. Gemcitabine significantly increased the surface area occupied by PANC1 cells (Fig. 5*C*). When *PLAUR* expression was silenced, the increase in the size of adherent cells, caused by gemcitabine, was significantly inhibited.

### scRNA-Seq identifies uPAR as a driver of malignant epithelial cell aggressiveness in human PDAC

**Figure 6:**
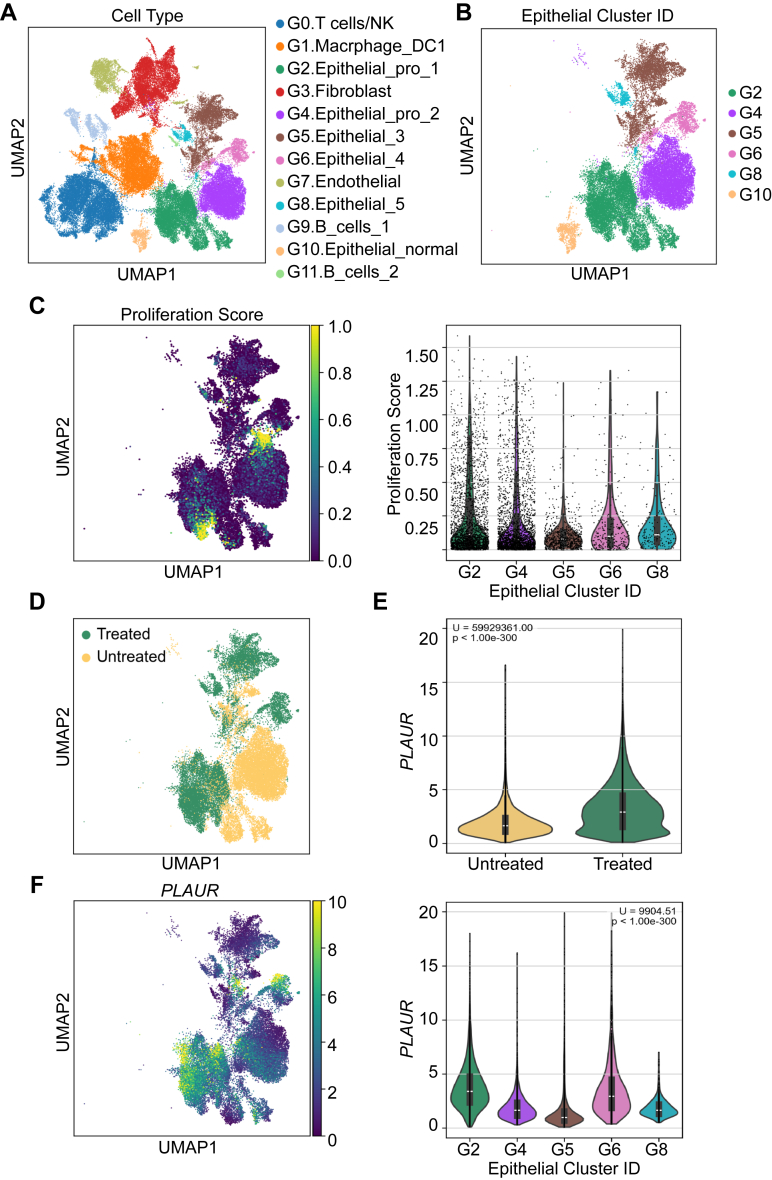
**scRNA-seq analysis reveals distinct epithelial cell populations in PDAC with differential *PLAUR* expression pre- and post-chemotherapy**. *A*, comprehensive UMAP visualization of single-cell transcriptomic data from six untreated and six chemotherapy-treated human PDAC samples, illustrating the major cell clusters identified. *B*, UMAP showing distinct epithelial cell clusters based on unique gene expression signatures. *C*, UMAP (*left*) and violin plots (*right*) illustrating proliferation scores across malignant epithelial cell clusters. *D*, UMAP plot showing the distribution of malignant epithelial cells from treated and untreated PDAC samples into various clusters. *E*, Violin plots comparing *PLAUR* expression in malignant epithelial cells in chemotherapy-treated and untreated patient samples. Cells in the various malignant epithelial cell clusters were analyzed collectively. *F*, UMAP (*left*) and Violin plots (*right*) displaying *PLAUR* expression in the various malignant epithelial cell clusters. Statistical significance was assessed by Mann-Whitney U and Kruskal-Wallis tests with Dunn's multiple comparisons.

scRNA-seq datasets, obtained by analyzing freshly collected human PDAC samples before and after chemotherapy (GSE205013) (51), were examined. Six untreated and six treated cases were analyzed together to generate a comprehensive UMAP, showing the major clusters of cells distinguished based on transcriptome signatures (Fig. 6*A*). Epithelial cells partitioned into six distinct clusters (Fig. 6*B*). One cluster, labeled G10, included epithelial cells with gene expression profiles indicative of non-tumorigenic, “normal” cells. This cluster was characterized by the lowest Copy Number Variation (CNV) score. CNV is an important biomarker, associated with genomic instability and increased tumorigenicity (52). By contrast, clusters G2, G4, G5, G6, and G8 consisted of malignant epithelial cells. Amongst these, the G2 and G4 clusters demonstrated the highest mean proliferative scores (Fig. 6*C*).

Cells in malignant epithelial cell clusters were stratified based on whether they were from treated or untreated specimens (Fig. 6*D*). Cluster G4 was composed almost entirely of untreated cells; this cluster essentially disappeared when only treated cells were examined. By contrast, the G2 cluster included treated as well as untreated cells, indicating that malignant epithelial cells in this cluster survived and may have expanded because of chemotherapy exposure.

We examined *PLAUR* expression in treated *versus* untreated cells, considering cells in all five malignant epithelial cell clusters together. *PLAUR* expression was significantly higher in cells from chemotherapy-treated samples *versus* untreated cells (Fig. 6*E*). A similar analysis was performed examining expression of *PLAU*. Like *PLAUR*, *PLAU* was expressed at significantly increased levels in treated malignant epithelial cells compared with untreated cells (Fig. S2*A*).

Next, we compared *PLAUR* expression across the five clusters of malignant epithelial cells, including both treated and untreated cells. *PLAUR* expression was significantly higher in cells in G2 compared with G4, G5, and G8 (Fig. 6*F*). *PLAUR* expression also was increased in cells in cluster G6, which, like G2, was composed largely of treated PDAC cells; however, G6 was a much smaller cluster than G2. Figure S2*B* shows that *PLAU* expression was highest in G2.

### Correlation of *PLAUR* expression with indices of PDAC progression

**Figure 7:**
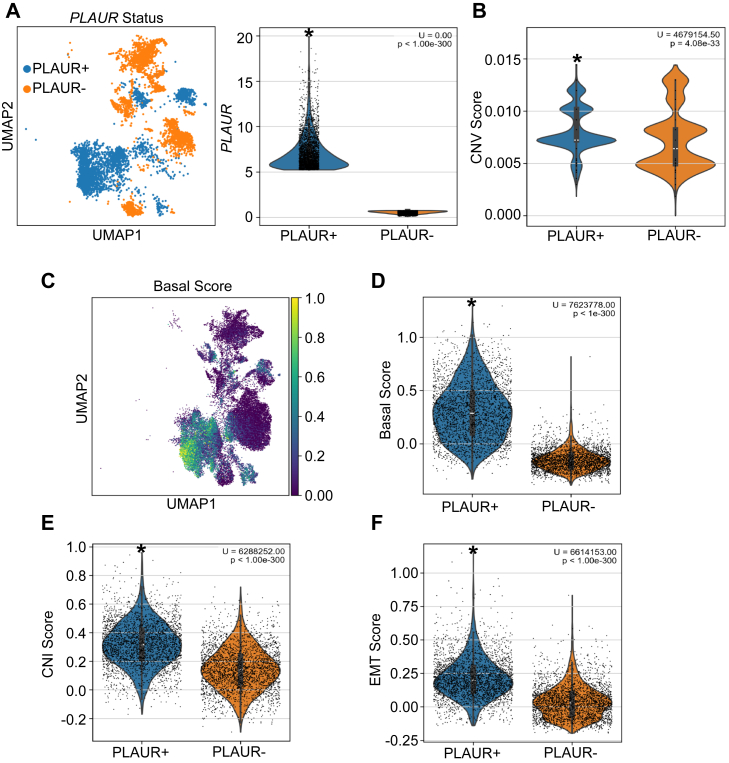
**Association of *PLAUR* expression in malignant epithelial cells with biomarkers of cancer aggressiveness**. *A*, UMAP (*left*) illustrating stratification of 32,118 malignant epithelial cells based on *PLAUR* expression into *PLAUR*-high (*PLAUR*+, *top* 10%) and *PLAUR*-low (*PLAUR*–, *bottom* 10%) groups. Violin plots (*right*) show *PLAUR* expression in these two groups. *B–F*, analyses comparing tumor-related phenotypes between *PLAUR*+ and *PLAUR*− cell populations. *B,* Violin plot comparing the CNV scores of *PLAUR*+ and *PLAUR*– malignant epithelial cell populations. *C,* UMAP representing the distribution of basal cell signature scores across epithelial cell clusters. *D*, Violin plots comparing the basal cell signature scores. *E*, CNI scores are compared. *F,* EMT signature scores are compared. Statistical significance (U-values and *p*-values) is shown in the figure (Kruskal–Wallis with Dunn's post-test and Mann–Whitney *U* test).

We stratified malignant epithelial cells in the composite (12 cases) PDAC UMAP into three groups based on *PLAUR* expression: high *PLAUR* (*PLAUR*+), intermediate, and low *PLAUR* (*PLAUR*−). Cells that expressed *PLAUR* in the top 10% were considered PLAUR+ and cells that expressed *PLAUR* in the bottom 10% were considered *PLAUR*−. Sorting of *PLAUR*+ and *PLAUR*− cells into the five malignant epithelial cell clusters is shown in Figure 7*A*. *PLAUR+* cells were associated largely with cluster G2.

*PLAUR+* cells demonstrated higher CNV scores compared with *PLAUR*-cells (Fig. 7*B*). The basal cell signature score was highest in the G2 cluster (Fig. 7*C*) and substantially increased in *PLAUR*+ malignant epithelial cells compared with *PLAUR*− cells (Fig. 7*D*). Basal cell-like PDAC cells are noted for high plasticity, mesenchymal traits, and poor differentiation (53).

Next, we calculated the Copy Number Instability (CNI) score, a measure of genomic instability that reflects the extent of chromosomal alterations in individual cells (52). *PLAUR*+ cells exhibited significantly higher CNI scores compared with *PLAUR*− cells (Fig. 7*E*). Finally, EMT signature analysis revealed significantly higher EMT scores in *PLAUR*+ *versus PLAUR*− cells (Fig. 7*F*). EMT is known to enhance tumor cell migration and invasiveness (54). The association of high *PLAUR* expression with increased EMT scores provides important confirmation of results obtained using cell culture model systems (24, 25, 55).

**Figure 8:**
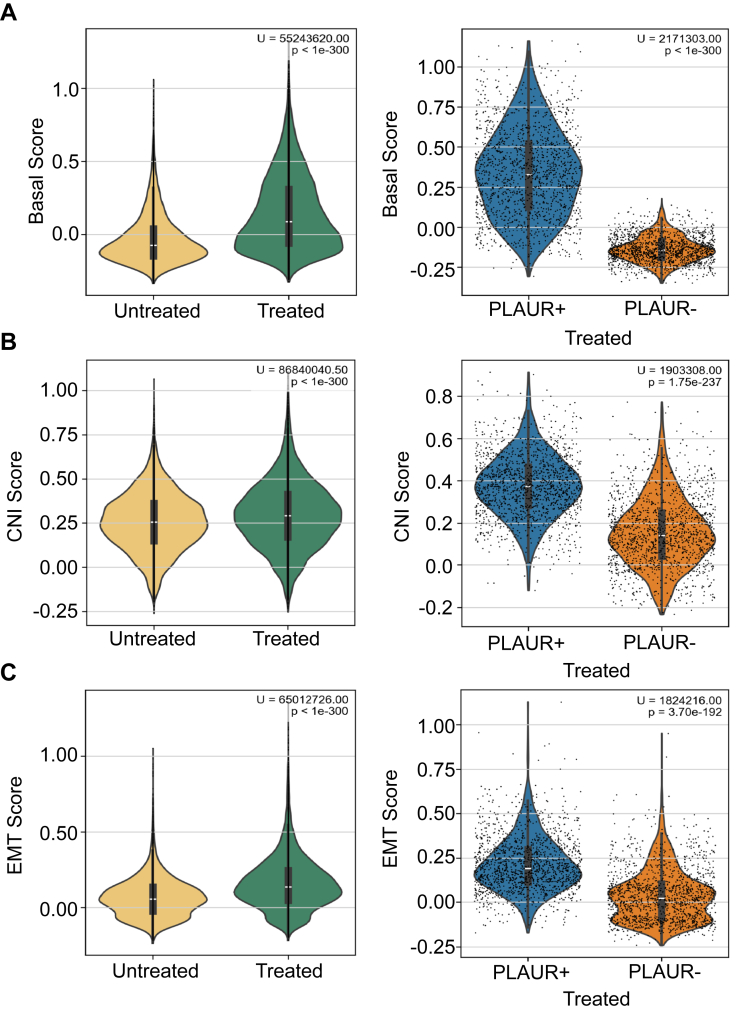
***PLAUR* expression correlates with basal, CNI, and EMT signatures in chemotherapy-treated PDAC malignant epithelial cells**. *A–C*, Violin plots comparing: *A,* basal cell signature scores; (*B*) CNI scores; and (*C*) EMT scores in treated *versus* untreated malignant epithelial cells (*left*) and in *PLAUR*-high (*PLAUR*+) *versus PLAUR*-low (*PLAUR*–) malignant epithelial cells (*right*). In the right-hand panels, only chemotherapy-treated PDAC cells were considered. Statistical significance (U-values and *p*-values) is shown in the figure (Kruskal–Wallis with Dunn's post-test and Mann–Whitney *U* test).

Next, basal cell signature scores, CNI scores, and EMT scores were calculated for *PLAUR*+ and *PLAUR*− malignant epithelial cells, considering cells from treated and untreated PDAC specimens separately. Figure 8 shows that all three scores were significantly increased in the treated *versus* untreated PDAC specimens. Among treated PDAC cells, all three scores were higher in the *PLAUR+* population, compared with the *PLAUR*− population (Fig. 8). When the identical analysis was performed, selectively examining untreated PDAC specimens, *PLAUR*+ cells still exhibited higher basal, CNI, and EMT scores compared with *PLAUR*− cells (Fig. S3).

## Discussion

*TP53* GoF mutations are common in PDAC (3); however, the molecular mechanisms by which these mutations promote cancer progression remain incompletely understood. Herein, we defined a novel relationship between *TP53* and the cancer-promoting receptor, uPAR, which explains many of the activities of *TP53* GoF mutations. In PANC1 and MIA PaCa-2 cells, which carry distinct *TP53* mutations, gemcitabine treatment significantly increased *PLAUR* expression, and this response was dependent on *TP53*. The role of *TP53* in controlling *PLAUR* expression was confirmed in human PDAC cases by mining TCGA. The potential for chemotherapy to increase *PLAUR* expression in malignant epithelial cells was confirmed by analyzing scRNA-Seq data.

The increase in *PLAUR* expression caused by gemcitabine may reflect the function of uPAR as a major component of a cell stress-survival system. Based on its ability to activate ERK1/2 and associated anti-apoptotic cell-signaling pathways, uPAR has been shown to demonstrate pro-survival activity in diverse cancer cells (16, 22, 29, 55, 56). uPAR expression is increased in breast cancer cells exposed to hypoxia *in vitro* (25). uPAR may confer resistance to cisplatin in squamous cell carcinoma of the head and neck (57, 58). uPAR also may promote survival of estrogen-dependent breast cancer cells when estrogen is withdrawn (59). When the constitutively active EGF receptor variant, EGFRvIII, is targeted with tyrosine kinase inhibitors in glioblastoma, increased expression of uPA is observed; the uPA binds to uPAR, activating uPAR-dependent cell signaling, which promotes cell survival in the absence of functional EGFRvIII (29, 60).

In our analyses of human PDAC scRNA-Seq data, two clusters of malignant epithelial cells emerged as having the highest proliferative indices, G2 and G4. Of the two clusters, *PLAUR* expression was significantly higher in G2. G4 was essentially eliminated by PDAC treatment, whereas G2 remained unchanged or may have been augmented. Because G2 demonstrated the highest level of *PLAUR* expression, these data are consistent with our model in which uPAR promotes cancer cell survival under challenging conditions. The cells in G4 were either killed in therapy or underwent substantial changes in gene expression so that the cells relocalized to other clusters.

There is substantial overlap between the cell-signaling pathways that promote cell survival and those that facilitate processes associated with cancer progression. In multiple cell culture and animal model systems, uPAR promotes cancer cell invasion and metastasis (17, 19, 61, 62). We previously demonstrated that uPAR promotes EMT in hypoxic breast cancer cells and, inversely, that *PLAUR* gene-silencing induces mesenchymal-epithelial transition (24). We now show by analysis of scRNA-Seq data that in PDAC, malignant epithelial cells with the highest levels of *PLAUR* mRNA have the highest EMT gene expression signatures. Thus, the association of uPAR with EMT has been validated in human tumors.

In PANC1 and MIA PaCa-2 cells, the increase in abundance of uPAR induced by gemcitabine was associated with substantial increases in phospho-ERK1/2. This increase was blocked by silencing *PLAUR* or *TP53*. p-RSK was also increased in gemcitabine-treated PDAC cells in a uPAR-dependent manner. RSK is a pro-survival cell-signaling factor known to be regulated by activated ERK1/2 (42, 63). The gemcitabine-induced increase in uPAR abundance promoted PDAC cell migration and invasion. Silencing *PLAUR* inhibited the effects of gemcitabine on cell migration and invasion. These results suggest that regulating p53 may represent an important approach for controlling the effects of uPAR on cancer progression.

Mechanisms by which uPAR may facilitate cancer invasion and metastasis span the plasma membrane. On the cell surface, binding of uPA to uPAR increases the catalytic efficiency of plasminogen activation. Once plasmin is generated, it compromises barriers to cell migration and invasion, activates growth factors, and remodels the extracellular matrix (61). The cell-signaling pathways activated by uPA-binding to uPAR are linked directly to invasion, metastasis, EMT, and expression of cancer stem cell-like properties (16, 24, 29, 56). In TCGA, high *PLAU* expression, like *PLAUR* expression, was associated with poorer survival indices in PDAC. In scRNA-Seq analyses, *PLAU* mRNA was increased in treated PDAC cells. Furthermore, the G2 malignant cell cluster, which included cells that survived treatment, had the highest level of *PLAU* mRNA. These results support a model in which uPA-binding to uPAR is critical for activation of its cancer-promoting activities. Furthermore, the malignant epithelial cells that express uPAR and are thus targets for tumor-associated uPA also may be the source of the uPA, so that activation of uPAR-dependent cell-signaling occurs *via* an autocrine pathway.

Mutated p53 regulates EMT by increasing expression of TWIST1 and ZEB1 (64, 65, 66, 67), promotes cancer invasion and metastasis (6, 67, 68), and regulates RhoA (69). Mutated p53 also up-regulates PDGFRβ expression, facilitating tumor cell dissemination and metastasis to the lung (70). Our phalloidin-staining data suggest that uPAR may be an effector of the reported effects of mutated p53 on RhoA activity. Understanding the role of uPAR as a mediator in individual molecular events ascribed to mutated p53 is an important future goal.

## Experimental procedures

### Cell lines and transfection

The human PDAC cell lines, MIA PaCa-2 and PANC1, were purchased from and validated by the ATCC (CRL-1420, RRID:CVCL_0428, and CRL-1469, RRID:CVCL_0480). They were routinely tested for *mycoplasma* contamination. Cultures were passaged no more than 10 times. Cells were cultured in DMEM with high glucose supplemented with 10% fetal bovine serum (FBS), 2.5% horse serum, and 1% penicillin-streptomycin.

Gene silencing was performed using Lipofectamine RNAiMAX. Cells were transfected with Silencer Select siRNA targeting *PLAUR* (80 nM; ID: s10615), *TP53* (50 nM; ID: s607), *EGFR* (50 nM; ID: s565) or Silencer Select Negative Control No. 1 siRNA (siCNTRL) from Thermo Fisher Scientific. In separate studies, *PLAUR* was silenced using a pool of four different siRNAs targeting distinct regions of the *PLAUR* gene (ON-TARGET plus human *PLAUR* siRNA SMARTpool; Cat# L-006388-00-0005; ID: 5329) from Horizon Discovery. siRNA sequences are available on the manufacturers' websites. Experiments were performed 24 to 48 h after gene silencing.

### Immunoblot analysis

Cells were extracted into RIPA buffer (Sigma-Aldrich) supplemented with protease inhibitors and phosphatase inhibitors (Thermo Fisher Scientific). Protein concentrations were determined using the bicinchoninic acid assay (Pierce Rapid Gold BCA Protein Assay Kit). Samples were resolved by SDS-PAGE and transferred to PVDF membranes. The membranes were blocked with 5% nonfat dry milk and incubated overnight with primary antibodies against uPAR (1:500, Cat# 12713, RRID:AB_2798003 and Cat# 12863, RRID:AB_2798050), β-Actin (1:20,000, Cat# 3700, RRID:AB_2242334), GAPDH (1:1,000, Cat# 97166, RRID:AB_2756824), p53 (1:1,000, Cat# 2527, RRID:AB_10695803), phosphorylated ERK1/2 (P-ERK1/2) (1:1,000, Cat# 4370, RRID:AB_2315112), total ERK1/2 (T-ERK1/2) (1:1,000, Cat# 9102, RRID:AB_330744), phosphorylated RSK (Thr 359) (1:1,000, Cat# 8753, RRID:AB_2783561) and EGF Receptor (1:1,000, Cat # 4267, RRID:AB_2246311) from Cell Signaling Technology. All primary antibodies were validated by Cell Signaling. After washing, membranes were incubated with secondary antibodies (Jackson ImmunoResearch Labs Cat# 115–035–146, RRID:AB_2307392 and Cat# 111–035–144, RRID:AB_2307391) and imaged by enhanced chemiluminescence using the Azure Biosystems c300 system or the Uvitec Alliance Q9-series imagers. Densitometry analysis was performed and the relative intensity of specific bands on each immunoblot was determined using ImageJ.

### Gene expression analysis by RT-qPCR

RNA was extracted using the NucleoSpin RNA kit (Macherey-Nagel) and reverse-transcribed using the iScript cDNA synthesis kit (Bio-Rad). RT-qPCR was performed using TaqMan Gene Expression Assays for human *PLAUR* (Hs00958880_m1) and *18S* rRNA (Hs99999901_s1) in a QuantStudio 3 Real Time PCR System (RRID:SCR_018712). The relative change in mRNA expression was calculated using the 2^ΔΔCt^ method.

### Cell migration assays

Bidirectional cell migration into 500 μM preformed gaps in monolayer culture was studied using the Ibidi USA Culture-Insert 2 Well for Self-Insertion system. Cells were transfected with siRNA targeting *PLAUR* or with siCNTRL. 24 h later, 10^4^ cells were plated into each well, with inserts in place, and treated with gemcitabine (100 μM; Sigma-Aldrich G6423-10 MG) or vehicle for 48 h. The inserts were then removed, and cell migration was allowed to proceed for 24 h. Images of the identical gap areas were acquired at 0 and 24 h. The percentage of the gap filled by migrating cells was calculated.

### Invasion assays

Cell invasion assays were performed using 6.5 mm Transwell inserts with polycarbonate membranes and 8.0 μm pores (Corning). Geltrex-reduced Growth Factor Basement Membrane Matrix (Thermo Fisher Scientific) was reconstituted in the upper wells (100 μl/well). PANC1 cells were transfected with siRNA targeting *PLAUR* or with siCNTRL and then treated for 48 h with gemcitabine (100 μM) or vehicle. 10^5^ cells were seeded into the upper chambers. The bottom chambers contained 10% FBS as a chemoattractant. Cells were allowed to invade for 36 h. Invaded cells on the underside of each membrane were fixed and stained using the PROTOCOL Hema 3 Manual Staining System and Stat Pack (Fisher HealthCare). Stained cells were visualized under a light microscope. Each condition was performed in technical duplicates, and at least four images per well were acquired. Cells were counted using ImageJ (RRID:SCR_003070) and the fold increase in invasion was calculated relative to that observed with cells transfected with siCNTRL. Each experiment was repeated four independent times with separate cultures.

### MTT assays

Cell viability was assessed using the Cell Proliferation Kit I (Roche). MIA PaCa-2 and PANC1 cells were seeded at 8000 to 10,000 cells per well into 96-well plates. The following day, cells were treated with gemcitabine and incubated for 24 to 72 h. MTT reagent (0.5 mg/ml) was added to each well for 4 h. Solubilization buffer (100 μl) was then added. Absorbance was measured at 570 nm and 650 nm; final readings were obtained by subtracting the 650 nm absorbance from the 570 nm measurement. Viable cells were calculated relative to time zero (prior to adding gemcitabine). Each experiment was performed in technical triplicate and repeated six independent times.

### Analysis of adherent cell surface area by immunofluorescence microscopy

PANC1 cells were plated on coverslips, transfected with siRNA targeting *PLAUR* or with siCNTRL, and incubated for 24 h before treatment with 100 μM gemcitabine or vehicle for 48 h. Cells were fixed with 4% PFA, permeabilized with 0.5% Triton X-100, and then stained using Flash Phalloidin Green 488 (BioLegend, 1:100). Slides were mounted using ProLong Gold Antifade Mountant with DAPI (Thermo Fisher Scientific). Fluorescence images were acquired using a Leica DMi8 microscope (RRID:SCR_026672). The surface area of 100 randomly selected cells in each slide was determined using ImageJ (RRID:SCR_003070). The experiment was replicated four times using separate cultures.

### Bioinformatics analyses

To compare the expression of *PLAUR* and *PLAU* in PDAC and adjacent normal tissues, we used GEPIA. Data from TCGA (The Cancer Genome Atlas, RRID:SCR_003193) were mined *via* cBioPortal (RRID:SCR_014555). PDACs were divided into two groups: those with missense mutations (n = 60 for *PLAUR*, n = 62 for *PLAU*) and those with wild-type *TP53* (n = 63 for *PLAUR*, n = 66 for *PLAU*). Kaplan-Meier survival graphs report OS, DFS, and PFS. Tumors with expression on *PLAUR* or *PLA*U in the top 25% were compared with those in the bottom 25%.

### scRNA-seq

scRNA-seq data from human PDACs, including untreated and treated primary specimens (n = 6 of each) were analyzed. Data were retrieved from the Gene Expression Omnibus (GEO, RRID:SCR_005012) Series GSE205013 (51).

Unprocessed.mtx files were pooled into our analysis pipeline built using scanpy (RRID:SCR_018139) (71) and scVI-tools (RRID:SCR_026673) (72). Cells expressing fewer than 200 genes and genes detected in fewer than 10 cells were excluded. Doublets were identified and removed using the SOLO model from the scVI package. Cells with mitochondrial and ribosomal gene counts exceeding more than 15% of the total gene pool were removed. Cells with abnormally high gene counts, above a 98th percentile threshold were also excluded.

Following sample processing, ribosomal genes were removed to enhance the detection of biologically relevant expression patterns. Batch correction and normalization were applied using an scVI-trained model. The library-sized normalized gene values were then used to calculate nearest-neighbors, and the Leiden algorithm (resolution = 0.1) was applied for clustering, followed by Uniform Manifold Approximation Projection (UMAP) visualization. Initial automated cluster annotation was performed using the CellTypist package (RRID:SCR_024893, https://www.celltypist.org/) with the 'Immune_All_Low.pkl' model (73). The rank_genes_groups function in scanpy was used to identify the top marker genes for each cluster. After assigning predicted labels, cluster annotations were finalized through manual verification using known marker genes from PanglaoDB (RRID:SCR_022580) (74) (Fig. S4). As an additional filtering step, only cells labeled as “epithelial cells” by CellTypist's *majority_voting* prediction from the malignant UMAP clusters were retained for downstream analysis.

Following identification of tumor epithelial cells, samples were stratified by treatment status and by *PLAUR*/*PLAU* expression. *PLAUR*/*PLAU* expression was examined within each UMAP-defined epithelial cluster, with differences validated through a Kruskal-Wallis test and Dunn's *post hoc* analysis. Analyses were performed in which all epithelial cells were considered together and in which treated *versus* untreated cells were considered separately. The top 10 percent (*PLAUR*+) and bottom 10 percent (*PLAUR*−) of cells were selected for analysis. The resulting final cohorts included 2813 cells for both groups (high and low *PLAUR* expression) across all conditions, and n = 1499 treated *versus* n = 1314 untreated cells. Gene set scoring using the scanpy *score_genes* function was used to assess differences in proliferation, EMT, CNI, and basal cell-type classification (Table S1). Distributions of basal, EMT, and CNI scores for *PLAUR+* and *PLAUR*− cells were visualized as violin plots. Mann-Whitney U-statistics were applied to determine the statistical significance of differences noted in *PLAUR+* and *PLAUR*− populations.

CNV analysis was performed on the epithelial cell subsets using inferCNV (RRID:SCR_021140, https://github.com/broadinstitute/inferCNV), with the immune cell population designated as the normal reference to establish baseline gene expression. A sliding window of 250 genes was applied to calculate CNV scores on a per-cell basis. The lowest CNV-scoring epithelial cell cluster, representing normal-adjacent tissue, was removed for downstream analysis.

### Statistics

Statistical analyses were performed using GraphPad Prism 10.0 (RRID:SCR_002798, GraphPad Software Inc.). All data are expressed as the mean ± standard error of the mean (SEM). Comparisons between two groups were performed using two-tailed unpaired t-tests. For comparisons involving more than two groups, one-way ANOVA or two-way ANOVA was used. For Kaplan-Meier survival analyses, significance was assessed using the log-rank Mantel-Cox test. *p*-values less than 0.05 were considered statistically significant (∗*p* < 0.05; ∗∗*p* < 0.01; ∗∗∗*p* < 0.001; ∗∗∗∗*p* < 0.0001).

## Data availability

The clinical data for TCGA-PAAD cohort were obtained from TCGA using GEPIA 2 (RRID:SCR_018294) and cBioPortal (RRID:SCR_014555). scRNA-seq data analyzed in this study were obtained from GEO database (RRID:SCR_005012) at GSE205013. The used code is deposited in Code Ocean (RRID:SCR_015532; https://doi.org/10.24433/CO.9170906.v1). All other raw data that support the findings of this study are available from the corresponding author upon reasonable request.

## Supporting information

This article contains supporting information.

## Conflict of interest

The authors declare that they have no conflicts of interest with the contents of this article.

## References

[1] Kamisawa T., Wood L.D., Itoi T., Takaori K. (2016). Pancreatic cancer. Lancet.

[2] Halbrook C.J., Lyssiotis C.A., Pasca di Magliano M., Maitra A. (2023). Pancreatic cancer: advances and challenges. Cell.

[3] Hezel A.F., Kimmelman A.C., Stanger B.Z., Bardeesy N., DePinho R.A. (2006). Genetics and biology of pancreatic ductal adenocarcinoma. Genes Dev..

[4] Pitolli C., Wang Y., Candi E., Shi Y., Melino G., Amelio I. (2019). p53-Mediated tumor suppression: DNA-damage response and alternative mechanisms. Cancers.

[5] Liu Y., Su Z., Tavana O., Gu W. (2024). Understanding the complexity of p53 in a new era of tumor suppression. Cancer Cell.

[6] Alvarado-Ortiz E., de la Cruz-López K.G., Becerril-Rico J., Sarabia-Sánchez M.A., Ortiz-Sánchez E., García-Carrancá A. (2021). Mutant p53 Gain-of-Function: role in cancer development, progression, and therapeutic approaches. Front. Cell Dev. Biol..

[7] Fiorini C., Cordani M., Padroni C., Blandino G., Di Agostino S., Donadelli M. (2015). Mutant p53 stimulates chemoresistance of pancreatic adenocarcinoma cells to gemcitabine. Biochim. Biophys. Acta.

[8] Zampieri C., Panatta E., Corbo V., Mauriello A., Melino G., Amelio I. (2022). p53 mutations define the chromatin landscape to confer drug tolerance in pancreatic cancer. Mol. Oncol..

[9] Mantovani F., Collavin L., Del Sal G. (2019). Mutant p53 as a guardian of the cancer cell. Cell Death Differ..

[10] Pfister N.T., Fomin V., Regunath K., Zhou J.Y., Zhou W., Silwal-Pandit L. (2015). Mutant p53 cooperates with the SWI/SNF chromatin remodeling complex to regulate VEGFR2 in breast cancer cells. Genes Dev..

[11] Kadosh E., Snir-Alkalay I., Venkatachalam A., May S., Lasry A., Elyada E. (2020). The gut microbiome switches mutant p53 from tumour-suppressive to oncogenic. Nature.

[12] Caporali S., Butera A., Ruzza A., Zampieri C., Bantula' M., Scharsich S. (2024). Selective metabolic regulations by p53 mutant variants in pancreatic cancer. J. Exp. Clin. Cancer Res..

[13] Do P.M., Varanasi L., Fan S., Li C., Kubacka I., Newman V. (2012). Mutant p53 cooperates with ETS2 to promote etoposide resistance. Genes Dev..

[14] Peart M.J., Prives C. (2006). Mutant p53 gain of function: the NF-Y connection. Cancer Cell.

[15] Cooks T., Pateras I.S., Tarcic O., Solomon H., Schetter A.J., Wilder S. (2013). Mutant p53 prolongs NF-κB activation and promotes chronic inflammation and inflammation-associated colorectal cancer. Cancer Cell.

[16] Gonias S.L. (2021). Plasminogen activator receptor assemblies in cell signaling, innate immunity, and inflammation. Am. J. Physiol. Cell Physiol..

[17] Andreasen∗ P.A., Egelund R., Petersen H.H. (2000). The plasminogen activation system in tumor growth, invasion, and metastasis. Cell. Mol. Life Sci..

[18] Smith H.W., Marshall C.J. (2010). Regulation of cell signalling by uPAR. Nat. Rev. Mol. Cell Biol..

[19] Blasi F., Carmeliet P. (2002). uPAR: a versatile signalling orchestrator. Nat. Rev. Mol. Cell Biol..

[20] Alfano D., Franco P., Stoppelli M.P. (2022). Modulation of cellular function by the urokinase receptor signalling: a mechanistic view. Front. Cell Dev. Biol..

[21] Mahmood N., Mihalcioiu C., Rabbani S.A. (2018). Multifaceted role of the urokinase-type plasminogen activator (uPA) and its receptor (uPAR): Diagnostic, prognostic, and therapeutic applications. Front. Oncol..

[22] Ma Z., Webb D.J., Jo M., Gonias S.L. (2001). Endogenously produced urokinase-type plasminogen activator is a major determinant of the basal level of activated ERK/MAP kinase and prevents apoptosis in MDA-MB-231 breast cancer cells. J. Cell Sci..

[23] Mauro C.D., Pesapane A., Formisano L., Rosa R., D'Amato V., Ciciola P. (2017). Urokinase-type plasminogen activator receptor (uPAR) expression enhances invasion and metastasis in RAS mutated tumors. Sci. Rep..

[24] Jo M., Lester R.D., Montel V., Eastman B., Takimoto S., Gonias S.L. (2009). Reversibility of epithelial-mesenchymal transition (EMT) induced in breast cancer cells by activation of urokinase receptor-dependent cell signaling. J. Biol. Chem..

[25] Lester R.D., Jo M., Montel V., Takimoto S., Gonias S.L. (2007). uPAR induces epithelial–mesenchymal transition in hypoxic breast cancer cells. J. Cell Biol..

[26] Jo M., Eastman B.M., Webb D.L., Stoletov K., Klemke R., Gonias S.L. (2010). Cell signaling by urokinase-type plasminogen activator receptor induces stem cell-like properties in breast cancer cells. Cancer Res..

[27] Kenny H.A., Leonhardt P., Ladanyi A., Yamada S.D., Montag A., Im H.K. (2011). Targeting the urokinase plasminogen activator receptor inhibits ovarian cancer metastasis. Clin. Cancer Res..

[28] Biagioni A., Chillà A., Del Rosso M., Fibbi G., Scavone F., Andreucci E. (2021). CRISPR/Cas9 uPAR gene knockout results in tumor growth inhibition, EGFR downregulation and induction of stemness markers in melanoma and Colon carcinoma cell lines. Front. Oncol..

[29] Wykosky J., Hu J., Gomez G.G., Taylor T., Villa G.R., Pizzo D. (2015). A urokinase receptor–bim signaling axis emerges during EGFR inhibitor resistance in mutant EGFR glioblastoma. Cancer Res..

[30] Gonias S.L., Hu J. (2015). Urokinase receptor and resistance to targeted anticancer agents. Front. Pharmacol..

[31] Zhai B.-T., Tian H., Sun J., Zou J.-B., Zhang X.-F., Cheng J.-X. (2022). Urokinase-type plasminogen activator receptor (uPAR) as a therapeutic target in cancer. J. Translational Med..

[32] Vial E., Sahai E., Marshall C.J. (2003). ERK-MAPK signaling coordinately regulates activity of Rac1 and RhoA for tumor cell motility. Cancer Cell.

[33] Aguirre-Ghiso J.A., Estrada Y., Liu D., Ossowski L. (2003). ERKMAPK activity as a determinant of tumor growth and dormancy; regulation by p38SAPK1. Cancer Res..

[34] Kastenhuber E.R., Lowe S.W. (2017). Putting p53 in context. Cell.

[35] de Geus S.W., Baart V.M., Boonstra M.C., Kuppen P.J., Prevoo H.A., Mazar A.P. (2017). Prognostic impact of urokinase plasminogen activator receptor expression in pancreatic cancer: malignant versus stromal cells. Biomark Insights.

[36] Peng L., Li Y., Yao S., Gaedcke J., Baart V.M., Sier C.F.M. (2023). Urokinase-type plasminogen activator receptor (uPAR) cooperates with mutated KRAS in regulating cellular plasticity and gemcitabine response in pancreatic adenocarcinomas. Cancers (Basel).

[37] Muller P.A.J., Vousden K.H. (2014). Mutant p53 in cancer: new functions and therapeutic opportunities. Cancer Cell.

[38] Xiong S., Chachad D., Zhang Y., Gencel-Augusto J., Sirito M., Pant V. (2022). Differential gain-of-function activity of three p53 hotspot mutants in vivo. Cancer Res..

[39] Lee S., Rauch J., Kolch W. (2020). Targeting MAPK signaling in cancer: mechanisms of drug resistance and sensitivity. Int. J. Mol. Sci..

[40] Samatar A.A., Poulikakos P.I. (2014). Targeting RAS-ERK signalling in cancer: promises and challenges. Nat. Rev. Drug Discov..

[41] Dalby K.N., Morrice N., Caudwell F.B., Avruch J., Cohen P. (1998). Identification of regulatory phosphorylation sites in mitogen-activated protein kinase (MAPK)-activated protein Kinase-1a/p90 rsk that are inducible by MAPK. J. Biol. Chem..

[42] Anjum R., Blenis J. (2008). The RSK family of kinases: emerging roles in cellular signalling. Nat. Rev. Mol. Cell Biol..

[43] Jost M., Huggett T.M., Kari C., Boise L.H., Rodeck U. (2001). Epidermal growth factor receptor-dependent control of keratinocyte survival and Bcl-xL expression through a MEK-dependent pathway∗. J. Biol. Chem..

[44] Liu D., Ghiso J.A.A., Estrada Y., Ossowski L. (2002). EGFR is a transducer of the urokinase receptor initiated signal that is required for in vivo growth of a human carcinoma. Cancer Cell.

[45] Jo M., Thomas K.S., O'Donnell D.M., Gonias S.L. (2003). Epidermal growth factor receptor-dependent and -independent cell-signaling pathways originating from the urokinase receptor. J. Biol. Chem..

[46] Fujita M., Imadome K., Imai T. (2017). Metabolic characterization of invaded cells of the pancreatic cancer cell line, PANC-1. Cancer Sci..

[47] Arora S., Bhardwaj A., Singh S., Srivastava S.K., McClellan S., Nirodi C.S. (2013). An undesired effect of chemotherapy: gemcitabine promotes pancreatic cancer cell invasiveness through reactive oxygen species-dependent, nuclear factor κB- and hypoxia-inducible factor 1α-MEDIATED up-regulation of CXCR4∗. J. Biol. Chem..

[48] Xu B.-Q., Fu Z.-G., Meng Y., Wu X.-Q., Wu B., Xu L. (2016). Gemcitabine enhances cell invasion via activating HAb18G/CD147-EGFR-pSTAT3 signaling. Oncotarget.

[49] Alfano D., Ragno P., Stoppelli M.P., Ridley A.J. (2012). RhoB regulates uPAR signalling. J. Cell Sci..

[50] Muller S.M., Okan E., Jones P. (2000). Regulation of urokinase receptor transcription by Ras- and rho-family GTPases. Biochem. Biophysical Res. Commun..

[51] Werba G., Weissinger D., Kawaler E.A., Zhao E., Kalfakakou D., Dhara S. (2023). Single-cell RNA sequencing reveals the effects of chemotherapy on human pancreatic adenocarcinoma and its tumor microenvironment. Nat. Commun..

[52] Yu M. (2011). Generation, function and diagnostic value of mitochondrial DNA copy number alterations in human cancers. Life Sci..

[53] Pitter K.L., Grbovic-Huezo O., Joost S., Singhal A., Blum M., Wu K. (2022). Systematic comparison of pancreatic ductal adenocarcinoma models identifies a conserved highly plastic basal cell state. Cancer Res..

[54] Liaghat M., Ferdousmakan S., Mortazavi S.H., Yahyazadeh S., Irani A., Banihashemi S. (2024). The impact of epithelial-mesenchymal transition (EMT) induced by metabolic processes and intracellular signaling pathways on chemo-resistance, metastasis, and recurrence in solid tumors. Cell Commun. Signal..

[55] Gilder A.S., Natali L., Van Dyk D.M., Zalfa C., Banki M.A., Pizzo D.P. (2018). The urokinase receptor induces a mesenchymal gene expression signature in glioblastoma cells and promotes tumor cell survival in neurospheres. Sci. Rep..

[56] Alfano D., Iaccarino I., Stoppelli M.P. (2006). Urokinase signaling through its receptor protects against anoikis by increasing BCL-xL expression levels. J. Biol. Chem..

[57] Cortes-Dericks L., Carboni G.L., Schmid R.A., Karoubi G. (2010). Putative cancer stem cells in malignant pleural mesothelioma show resistance to cisplatin and pemetrexed. Int. J. Oncol..

[58] Huang Z., Wang L., Wang Y., Zhuo Y., Li H., Chen J. (2013). Overexpression of CD147 contributes to the chemoresistance of head and neck squamous cell carcinoma cells. J. Oral Pathol. Med..

[59] Eastman B.M., Jo M., Webb D.L., Takimoto S., Gonias S.L. (2012). A transformation in the mechanism by which the urokinase receptor signals provides a selection advantage for estrogen receptor-expressing breast cancer cells in the absence of estrogen. Cell Signal..

[60] Hu J., Jo M., Cavenee W.K., Furnari F., VandenBerg S.R., Gonias S.L. (2011). Crosstalk between the urokinase-type plasminogen activator receptor and EGF receptor variant III supports survival and growth of glioblastoma cells. Proc. Natl. Acad. Sci. U. S. A..

[61] Bharadwaj A.G., Holloway R.W., Miller V.A., Waisman D.M. (2021). Plasmin and plasminogen system in the tumor microenvironment: implications for cancer diagnosis, prognosis, and therapy. Cancers.

[62] Minaei E., Mueller S.A., Ashford B., Thind A.S., Mitchell J., Perry J.R. (2022). Cancer progression gene expression profiling identifies the urokinase plasminogen activator receptor as a biomarker of metastasis in cutaneous squamous cell carcinoma. Front. Oncol..

[63] Sun Y., Tang L., Wu C., Wang J., Wang C. (2023). RSK inhibitors as potential anticancer agents: discovery, optimization, and challenges. Eur. J. Med. Chem..

[64] Chang C.-J., Chao C.-H., Xia W., Yang J.-Y., Xiong Y., Li C.-W. (2011). p53 regulates epithelial–mesenchymal transition and stem cell properties through modulating miRNAs. Nat. Cell Biol..

[65] Babaei G., Aliarab A., Asghari Vostakolaei M., Hotelchi M., Neisari R., Gholizadeh-Ghaleh Aziz S. (2021). Crosslink between p53 and metastasis: focus on epithelial-mesenchymal transition, cancer stem cell, angiogenesis, autophagy, and anoikis. Mol. Biol. Rep..

[66] Dong P., Karaayvaz M., Jia N., Kaneuchi M., Hamada J., Watari H. (2013). Mutant p53 gain-of-function induces epithelial-mesenchymal transition through modulation of the miR-130b-ZEB1 axis. Oncogene.

[67] Tang Q., Su Z., Gu W., Rustgi A.K. (2020). Mutant p53 on the path to metastasis. Trends Cancer.

[68] Schofield H.K., Zeller J., Espinoza C., Halbrook C.J., del Vecchio A., Magnuson B. (2018). Mutant p53^R270H^ drives altered metabolism and increased invasion in pancreatic ductal adenocarcinoma. JCI Insight.

[69] Timpson P., McGhee E.J., Morton J.P., von Kriegsheim A., Schwarz J.P., Karim S.A. (2011). Spatial regulation of RhoA activity during pancreatic cancer cell invasion driven by mutant p53. Cancer Res..

[70] Weissmueller S., Manchado E., Saborowski M., Morris J.P., Wagenblast E., Davis C.A. (2014). Mutant p53 drives pancreatic cancer metastasis through cell-autonomous PDGF receptor β signaling. Cell.

[71] Wolf F.A., Angerer P., Theis F.J. (2018). SCANPY: large-scale single-cell gene expression data analysis. Genome Biol..

[72] Gayoso A., Lopez R., Xing G., Boyeau P., Valiollah Pour Amiri V., Hong J. (2022). A python library for probabilistic analysis of single-cell omics data. Nat. Biotechnol..

[73] Xu C., Prete M., Webb S., Jardine L., Stewart B.J., Hoo R. (2023). Automatic cell-type harmonization and integration across human cell atlas datasets. Cell.

[74] Franzén O., Gan L.-M., Björkegren J.L.M. (2019). PanglaoDB: a web server for exploration of mouse and human single-cell RNA sequencing data. Database.

