# Supplementary material for: The urokinase receptor/uPAR is a major effector of p53 gain-of-function mutations in gemcitabine-treated pancreatic ductal adenocarcinoma
Source: J Biol Chem. 2025 Aug 7;301(9):110561. doi: 10.1016/j.jbc.2025.110561 (PMC12405631; doi:10.1016/j.jbc.2025.110561)
Supplement: Supplementary Material [file mmc1.pdf]

The urokinase receptor/uPAR is a major effector of p53 gain-of-function mutations in gemcitabine-treated pancreatic ductal adenocarcinoma

Carlotta Zampieri, Kourosh Kouhmareh, Parnian Sartipdolagh, Pardis Azmoon, Richard L. Klemke, and Steven L. Gonias

Supporting information

Supplementary Figure 1-4 and Supplementary Table 1

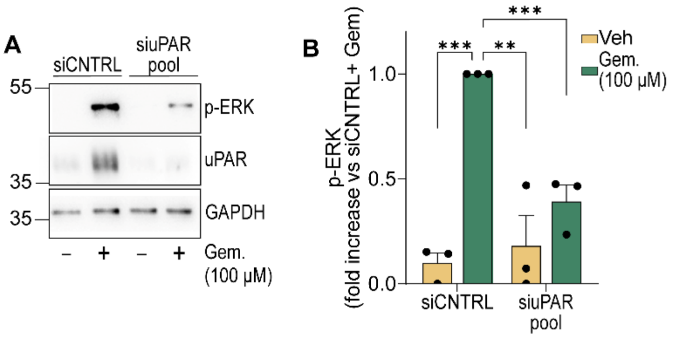

Supplementary Figure 1. A, Immunoblot analysis of p-ERK and uPAR in PANC1 cells transfected with control siRNA or with a pool of four PLAUR-specific siRNAs (siuPAR pool). Following transfection, the cells were treated with 100 µM gemcitabine or vehicle for 72 h. Immunoblots were re-probed for GAPDH as a loading control. **B**, Densitometric quantification of p-ERK levels normalized to GAPDH from three independent experiments. Data represent mean ± SEM (**P < 0.01; ***P < 0.001; one-way ANOVA and Dunnett's test)

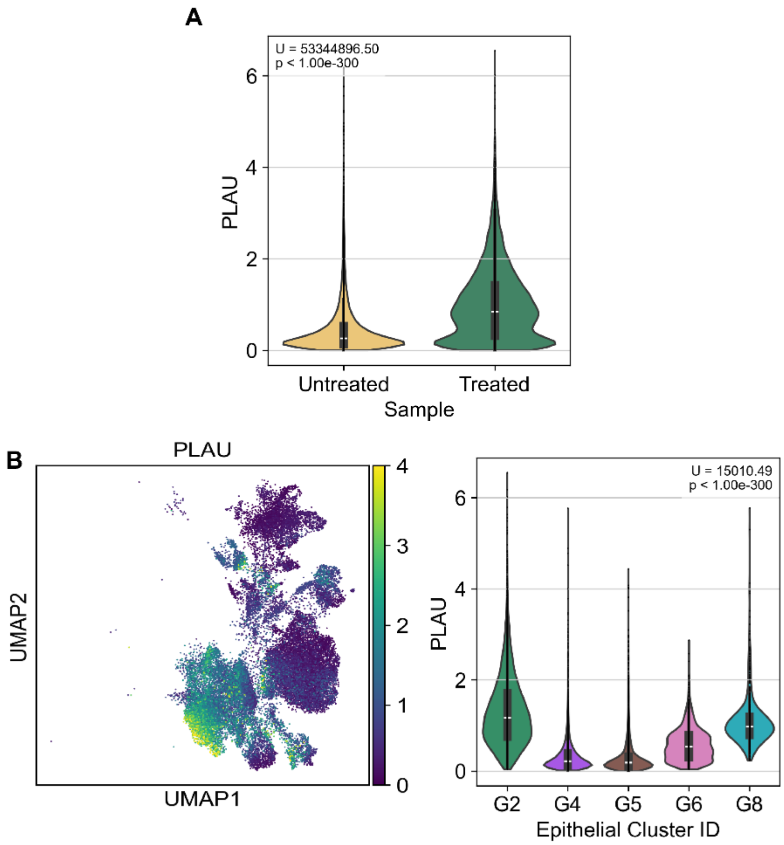

Supplementary Figure 2. A, Violin plots illustrating *PLAU* expression in malignant epithelial cells from chemotherapy-treated compared with untreated patient samples. **B,** UMAP (left) and violin plots (right) illustrating *PLAU* expression across distinct malignant epithelial cell clusters. Statistical analysis was performed using Mann-Whitney U and Kruskal-Wallis tests with Dunn's multiple comparisons.

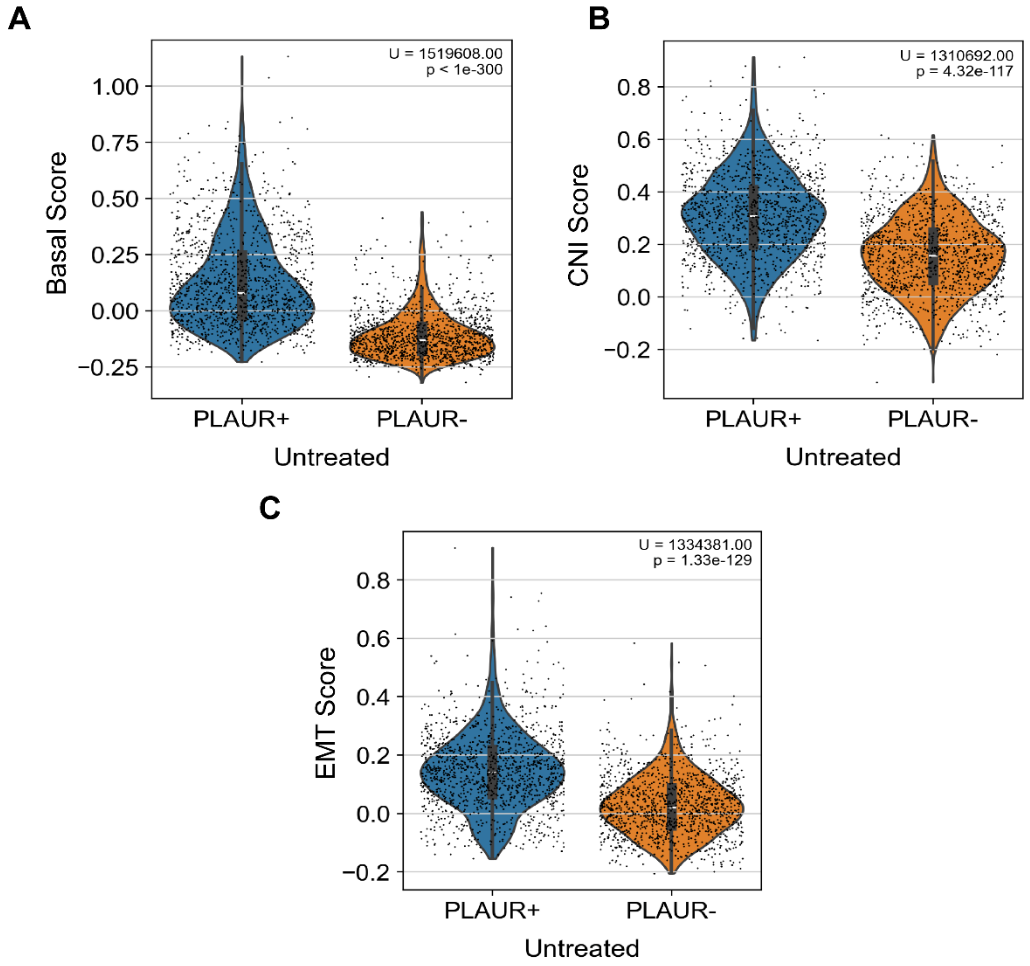

Supplementary Figure 3. *PLAUR* expression correlates with basal cell, CNI, and EMT signatures in untreated malignant PDAC epithelial cells. **A–C**, Violin plots comparing: **A,** basal cell signature scores; **B,** CNI scores; and **C,** EMT scores in *PLAUR*-high (*PLAUR*+) and *PLAUR*-low (*PLAUR*–) malignant epithelial cells from untreated PDAC specimens. Statistical significance (U-values and p-values) is shown in the figure (Kruskal–Wallis with Dunn's post-test and Mann–Whitney U test).

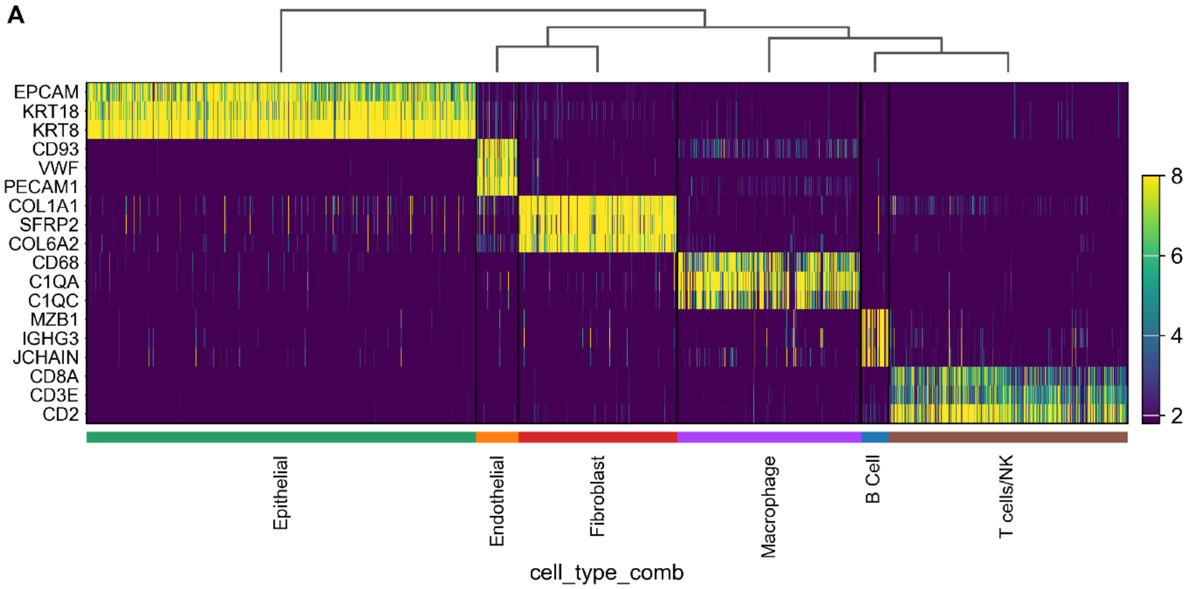

Supplementary Figure 4. Heatmap showing RNA-seq expression levels of marker genes characterizing the major cell clusters identified by scRNA-seq analysis. Columns represent individual cells grouped into epithelial, endothelial, fibroblast, macrophage, B cell, and T cell/NK cell populations based on transcriptomic profiles. Rows represent 18 differentially expressed marker genes specific for each cell type. The color scale represents the relative expression level of each gene across individual cells.

Signature	Genes list
Proliferation	*CDK1, MKI67, BUB1, MYBL2, PCNA, CCNB1, TOP2A, CCNA2, CDKN3, AURKA, AURKB, PLK1, TYMS, MCM3*
EMT	*CDH1, VIM, SNAI1, SNAI2, ZEB1, ZEB2, TWIST1, FN1, CDH2, MMP2, MMP9, ITGB1, ITGA5, FOXC2, GSC*
Basal PDAC Cell	*VGLL1, UCA1, S100A2, LY6D, SPRR3, SPRR1B, LEMD1, KRT15, CTSV, DHRS9, AREG, CST6, SERPINB3, KRT6A, SERPINB4, FAM83A, SCEL, FGFBP1, KRT7, KRT17, GPR87, TNS4, SLC2A1, ANXA8L1*
CNI	*EZH2, JUN, VIM, STEAP1, SOX4, MMP14, TIMP1, ZEB1, ITGB5, ITGB1, IGFBP4, FN1, DSC2, CXCL1, CTNNB1, BMP4, BCL2L1, KIF2A, SHH, KIF2C*

Supplementary Table 1. Gene sets used to assess differences in proliferation score, epithelial-mesenchymal transition (EMT), basal cell-type classification, and copy number instability (CNI).
